# Reflectance confocal microscopy of proliferative actinic keratoses: Two case reports

**DOI:** 10.1016/j.jdcr.2025.01.020

**Published:** 2025-02-13

**Authors:** Pedro Lobos, Carolina Gómez, Karol Baksai, Catalina Retamal

**Affiliations:** a Department of Dermatology, Clínica Las Condes, Santiago, Chile; b Department of Pathology, Clínica Las Condes, Santiago, Chile; c Department of Dermatology, Universidad de Chile, Santiago, Chile

**Keywords:** Actinic keratosis, confocal microscopy, proliferative actinic keratosis

## Clinical presentation

### Case 1

**Fig 1:**
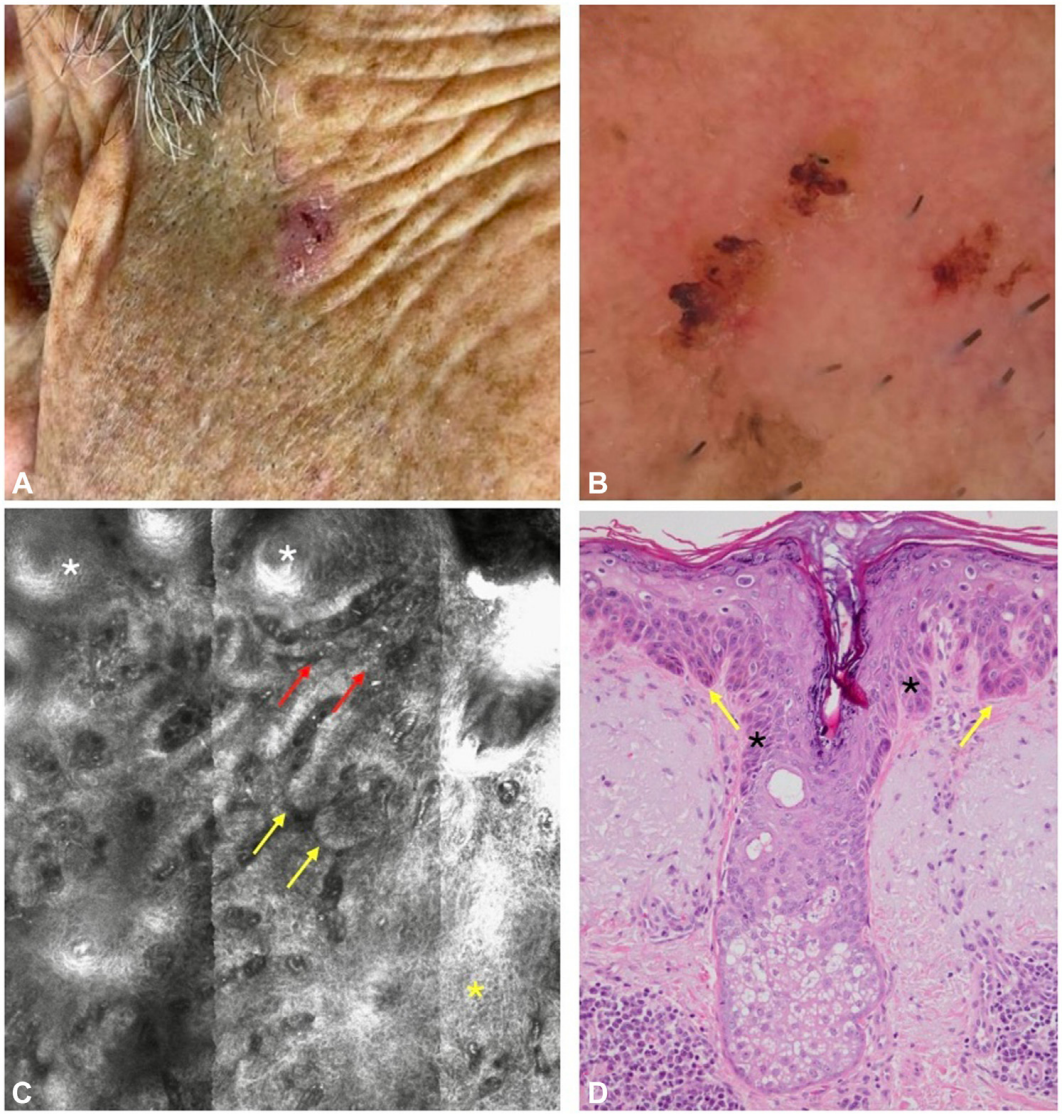
**A,** Clinical image: Red patch with scales and crusts on the right cheek. **B,** Dermatoscopy erythema with crusting and some targetoid ostia of hair follicles. **C,** An RCM image at the level of the suprabasal layer/papillary dermis shows an atypical honeycomb pattern and elongated structures and oval cords composed of atypical/pleomorphic cells (*yellow arrow*) with clefting, hair follicles (*white asterisk*), plump bright cells (*red arrow*), and atypical honeycomb pattern (*yellow asterisk*). **D,** Histopathology showed dermal projections of atypical keratinocytes (*yellow arrow*) affecting the infundibulum of the pilosebaceous unit (*asterisk*) (hematoxylin-eosin stain; original magnification: ×100).

A 65-year-old man presented with an erythematous asymptomatic slowly growing red patch with scales and crusts on the right cheek that appeared 1 year ago (Fig 1, *A*). A dermatoscopy revealed minimal background erythema with some targetoid ostia of hair follicles and crusting in some areas (Fig 1, *B*). A reflectance confocal microscopy (RCM) revealed an atypical honeycomb pattern in the epidermis. It also displayed multiple elongated protuberant extensions at the dermoepidermal junction resembling basal cell carcinoma (BCC) tumor islands with cleft-like spaces and melanophages (Fig 1, *C*). A histopathological examination revealed skin with severe actinic elastosis, irregular acanthosis, and dermal projections of atypical keratinocytes affecting the infundibulum of the pilosebaceous unit (Fig 1, *D*).

### Case 2

**Fig 2:**
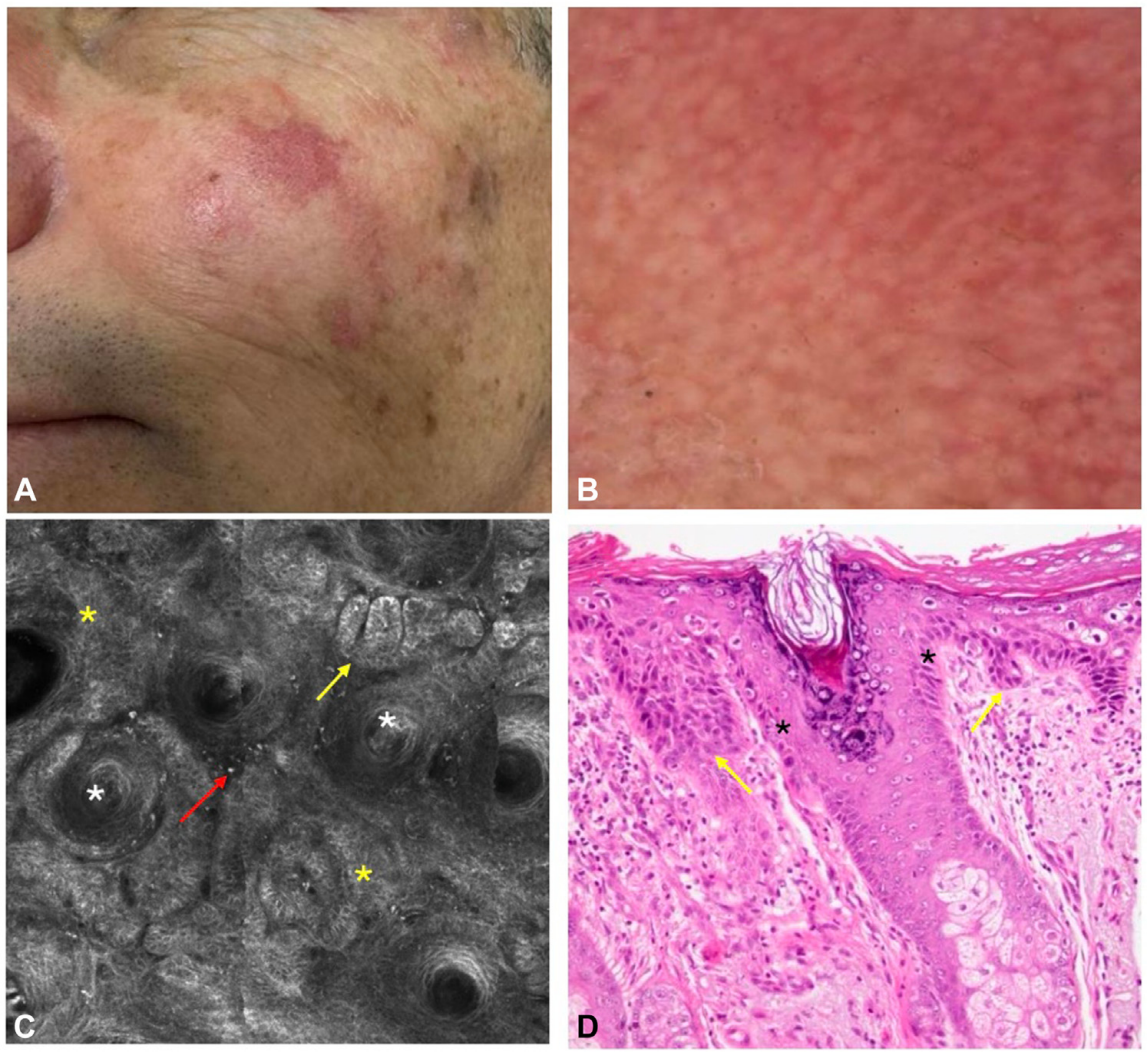
**A,** Clinical image: red patch on the left cheek. **B,** A dermatoscopy shows a faint reticular erythema and white circles. **C,** An RCM image at the level of the suprabasal layer/papillary dermis shows marked basal atypia and elongated and rounded cords (*yellow arrow*) with highly pleomorphic cells surrounding hair follicles (*white asterisk*) with cleft-like spaces, plump bright cells (*red arrow*), and atypical honeycomb pattern (*yellow asterisk*). **D,** A histopathology shows areas of bulbous prolongations of interpapillary processes composed of atypical keratinocytes (*yellow arrow*) near the follicular epithelium (*asterisk*; hematoxylin-eosin stain original magnification: ×40).

A 56-year-old man with a history of multiple actinic keratosis (AK) on the face presented with an extensive AK on the left cheek that appeared 9 months ago (Fig 2, *A*). A dermatoscopy showed subtle changes, including faint reticular erythema and white circles (Fig 2, *B*). An RCM demonstrated an atypical honeycomb pattern in the dermoepidermal junction and papillary dermis, with elongated oval cords surrounding the hair follicles. Cleft-like spaces and melanophages resembled a nodular BCC (Fig 2, *C*). Histopathology showed foci of parakeratosis, altered maturation of the lower third of the epidermis, and areas of bulbous prolongations of interpapillary processes that were adjacent to and occasionally affected the follicular infundibular epithelium (Fig 2, *D*).

## Discussion

AK has various subtypes that differ clinically and pathologically. Proliferative actinic keratosis (PAK) is an infrequent variant of AK that usually appears in patients with long-term sun exposure and severe actinic damage. This subtype is known to be resistant to standard therapies and to have an increased likelihood of developing invasive squamous cell carcinoma.^1^^,^^2^ PAK lesions often appear as scaly, erythematous macules or plaques with poorly defined borders, similar to our cases, and they can exceed 1 cm in size. Histologically, PAK is characterized by keratinocyte dysplasia in the lower third of the epidermis, with a downward growth pattern, papillary projections protruding into the dermis, and adnexal extension.^3^ An RCM has been validated as a diagnostic tool for AK that enables the visualization of parakeratosis with a disarranged epidermal pattern with atypical keratinocytes, densely packed round to polymorphous dermal papillae, polycyclic papillary contours, linear vessels, and coarse collagen bundles, to name more prominent features.^4^^,^^5^

However, the literature has not described the confocal features of PAK. We report 2 cases of AK that were referred to a confocal microscopy study to rule out BCC and squamous cell carcinoma. Both cases showed specific RCM findings of AK: an atypical honeycomb pattern and rare characteristics of PAK, including elongated bright tubular and oval structures comprising atypical keratinocytes extending into the dermis surrounding adnexal structures. This finding correlates with the histopathological view of areas of bulbous prolongations regarding the interpapillary processes of atypical keratinocytes that affect the follicular epithelium with cleft-like spaces and inflammatory cells (melanophages). This assists with the future management of this special subtype of AK. Caution must be exercised not to confound these findings with the bright tubular structures of solar lentigo (SL) that comprise regular keratinocytes connected to the dermal papilla. They do not follow the adnexal structures and tumoral islands of BCC that appear with tightly packed cells arranged in parallel and clefting typically surrounded by dilated linear and tortuous blood vessels, fibrotic tumor stroma, and occasionally dendritic cells. Table I shows the comparison of the different characteristics between SL, PAK, and BCC. Although more data are necessary to confirm our findings, we suggest that a PAK should be suspected when the RCM evaluation of a skin tumor with AK characteristics reveals: (1) elongated bright tubular and/or oval structures comprising atypical keratinocytes extending into the dermis with cleft-like spaces and inflammatory cells; (2) these structures are preferentially disposed to surrounding adnexal structures; and (3) the absence of RCM-based signs of SL and BCC.

**Table I:** Confocal features of SL, PAK, and BCC

Skin level	SL	PAK	BCC
Corneal layer	Normal	Parakeratosis	Normal
Spinous-granular level	Typical honeycomb pattern	Atypical honeycomb pattern	Typical honeycomb pattern, but can have areas of streaming of keratinocytes
Dermalepidermal junction/papillarydermis	Bright round cells at the suprabasal level, round to polycyclic edged papillae, cord-like rete ridges, and bulbous projections of normal and regular keratinocytes. Can have different degrees of solar elastosis	Pleomorphic cells in the basal layer, cords of atypical cells protruding into the dermis with adnexal extension, inflammatory cells, and an increase of vascularity. Severe solar elastosis	Tumor islands, cleft-like spaces, fibrotic tumor stroma, dilated and tortuous linear blood vessels, bright dendritic structures within the tumor islands, melanophages, and free melanin. Severe solar elastosis

*BCC*, basal cell carcinoma; *PAK*, proliferative actinic keratosis; *SL*, solar lentigo.

## Conflicts of interest

None disclosed.
